# The first experience with fully endoscopic posterior cervical foraminotomy and discectomy for radiculopathy performed in Viet Duc University Hospital

**DOI:** 10.1038/s41598-022-12493-x

**Published:** 2022-05-18

**Authors:** Son Ngoc Dinh, Hung The Dinh

**Affiliations:** 1 Faculty of Surgery, Ha Noi Medical University, 1 Ton That Tung Str., Dong Da Ha Noi, Viet Nam; 2 Spine Surgery Department, Viet Duc University Hospital, 40 Trang Thi Str., Hoan Kiem Ha Noi, Viet Nam

**Keywords:** Clinical trial design, Anatomy

## Abstract

The aim of the article is to present the first experience of applying a full-endoscopic posterior cervical foraminotomy and discectomy performed at Viet Duc University Hospital in Hanoi and describe the outcomes of such surgical intervention. This surgical series includes 20 patients underwent surgery through full-endoscopic posterior cervical foraminotomy and discectomy. The definitive diagnosis of the patients and the evidence for surgical treatment was radiculopathy due to lateral or intraforaminal disk herniation, foraminal stenosis, and lateral recess stenosis. Patients with discogenic cervical radiculopathy but with a contraindication to endoscopic posterior cervical foraminotomy and discectomy were not subject to surgical intervention. All patients underwent a CT and MRT examination of the cervical spine before and after surgery as complementary diagnostic methods. Besides radiological diagnostic methods, electroneuromyography and spondylography were performed with functional samples, i.e., with head tilts in the front and back, to eliminate segmental instability. The timing and degree of the root pain syndrome regression were assessed using a VAS scale (visual and analog scale) with a subsequent comparison of preoperative and postoperative performance. Immediately after the operation, all patients noted a complete or nearly complete regression of the pain syndrome.

## Introduction

The prevalence of discogenic compression syndromes at the cervical level is extremely high among people of working age and has a significant impact on their quality of life^1,2^. According to Lawrence^3^, among the total number of degenerative-dystrophic lesions of the spine, about 10% of the population suffer periodic compression pain in the cervical spine or the hand. A significant proportion of patients eventually need surgery^4^. The most common surgical methods in treating this pathology are anterior cervical discectomy and fusion (ACDF) and posterior cervical foraminotomy and discectomy (PCFD)^5,6^. At that, a posterior cervical foraminotomy is believed to be an effective technique that does not limit the range of motion in the cervical spine and minimizes the impact on degenerative changes in adjacent motor segments^7^. PCFD allows achieving adequate decompression and visualization of the outgoing nerve root in the region of the lateral recess and intervertebral aperture. In contrast to anterior discectomy, PCFD does not require stabilization^8,9^. Choosing the most optimal surgical treatment tactics for these pathologies is an urgent problem, which requires discussion. Minimal invasive PCFD has been used in clinical practice since the early 2000s^10,11^. The desire of specialists to minimize surgical aggression has influenced the evolution of endoscopic equipment, tools for effective bone resection and decompression, which predetermined the development of tube micro-endoscopy^12–14^ and percutaneous video endoscopy (full-endoscopic)^15–18^ in spine surgery. To date, a full-endoscopic method is the least invasive in spine surgery. Such operations on the lumbar spine have been proven to be safe and are performed routinely^19^. At the same time, the use of endoscopic methods in operations on the cervical spine is not so frequent. In 2007, Ruetten and co-authors^20^ reported on the experience of treating 87 patients with lateral hernias of the intervertebral discs of the cervical spine using a method of a full endoscopic PCFD with a 6.9 mm endoscope through a working cannula with an external diameter of 7.9 mm. Thus, the surgical intervention was performed using puncture access to the posterior structures of the cervical spine through a skin incision not exceeding 1 cm in length.

At present, some studies have been carried out to assess the efficiency of PCFD in comparison with the front percutaneous chiropractic endoscopic technique and the rear tube micro-endoscopy. The clinical results of the compared methods proved to be commensurate^6,21,22^. The authors point out the advantages of the full-endoscopic PCFD operative technique, which is the reproducibility of access, bright illumination of the operating cavity, and excellent visualization of anatomical structures in the liquid medium of the physiological solution of sodium chloride. Also, the possibilities of complete decompression of the spine under the targeted visual control have been established, which minimizes the risks of its damage, absence of necessity of stabilization due to complete safety of supporting structures, and no postoperative restrictions on the patient's activity^6,22,23^. However, the disadvantage of this technique is the inability to decompress the opposite part of the spinal canal and the technical complexity of the operation, which requires extensive experience of the surgeon in possession of not only microsurgical but also endoscopic techniques. When performing such interventions, the surgeon should be able to interpret the intraoperative radiological and video endoscopic picture as there are no indicators common for an open-access operation. Besides, such operations require the use of longer surgical instruments, which require working in cavities of small volume in conditions of constant irrigation with a physiological solution of sodium chloride, focusing on the video image at a changed angle of 25°–30° due to the form of the distal end of the endoscope. The above-described features of the method predetermine a long period of learning and the need for additional training of surgeons^24^.

Full-endoscopic surgery of the cervical spine has recently tended to be widespread. It is largely due to the general trends of modern spine surgery and the health care system as a whole, which implies the minimization of surgical interventions, reduction in the number of bed days, smaller consume of medications, and quick restore of patients` ability to work^25,26^.

However, in Vietnamese clinics, this surgical technique has not been used before.

Therefore, the study aimed to (i) report the first experience with fully endoscopic PCFD performed at Viet Duc University Hospital in Hanoi; (ii) identify the indications for such surgery; (iii) describe the technique and evaluate the results of this surgical intervention.

## Methods

### Study design and settings

A study design is a prospective uncontrolled cohort study describing a comprehensive surgical series of full-endoscopic posterior cervical surgery foraminotomy and discectomy (PCFD) first performed at the Department of Spine Surgery, Viet Duc University Hospital, Hanoi, Vietnam. Full endoscopy (PCFD) was conducted in 20 patients (12 males and 8 females, mean patient age was 51.87 ± 9.76 years) from February to September 2020. The group of patients included 18 primary surgery cases and 2 review cases. Two patients had previously undergone ACDF. After the operation, the development of radiculopathy caused by herniated intervertebral discs of the nerve roots was observed in patients, which resulted pronounced clinical symptoms. Afterward, PCFD was performed.

This study was approved by the Ethics Committee of Viet Duc University Hospital (Minutes No. 2614 of 23 January 2020) and conformed to the Helsinki Declaration. All patients signed informed consent forms.

### Eligibility criteria

Patients requiring full-endoscopic PCFD were selected according to preoperative diagnosis based on the established indications and contra-indications (inclusion and exclusion criteria) for such surgery.

During clinical examination, complaints of patients were recorded, anamnesis data were assessed, and neurological status of patients was evaluated. In addition to the standard assessment of the neurological status, all patients received a CT and MRT scan of the cervical spine. CT is preferable for the detection of compression factors (location of the hernia of the intervertebral disk, the number of bone osteophytes), while MRT is better in detecting the result of compression (spinal cord and roots compression). Also, it allows obtaining an image of the entire cervical spine and determining the degree of stenosis of holes.

The position of the intervertebral disc herniation and its density were determined based on the consolidated data. Afterward, the differential diagnostics of tumors of the spinal canal, traumas, inflammatory diseases, malformations, neuropathies, carpal tunnel syndrome, brachial periarthritis, and other dysfunctions was performed.

Radiography of the cervical spine in the straight and lateral projections was employed to assess the degree of the existing deformity. Spondylography with functional tests (head tilted forward and backward) was used to identify segmental instability. Cervical spine instability was interpreted as a condition in which the range of motions exceeded the physiological one (sagittal translation during flexion–extension greater than 3.5 mm, sagittal rotation during flexion greater than 11 degrees, positive posterior extension test, pathological reduction of intervertebral disc height). Instability was assessed according to the White-Panjabi criteria^27^.

The indications for full-endoscopic PCFD were as follows: unilateral monoradicular cervical pain syndrome due to lateral or foraminal ipsilateral single-level "soft" disc herniation in C2-C3 to C7-Th1 segments, secondary ipsilateral single-level foraminal stenosis with intervertebral joint hypertrophy.

### Inclusion and exclusion

Inclusion criteria: radiculopathy due to (i) lateral or intraforaminal disk herniation, (ii) foraminal stenosis, (iii) lateral recess stenosis.

Exclusion criteria: patients with discogenic cervical radiculopathy but having a contra-indication to endoscopic posterior cervical foraminotomy and discectomy: (i) axial neck pain; (ii) instability (5 and more scores according to White-Panjabi criteria); (iii) symptom due to central disc pathology; (iv) kyphotic deformity; (v) excessive ventral diseases.

### Intervention

#### Operating equipment

For surgical intervention, the endoscope Joimax (Germany) with an external diameter of 6.3 mm was employed. In addition to the optical system and fiber optic conductors, it contains an eccentrically located working channel for insertion of instruments with a diameter of 3.7 mm, as well as two channels with a diameter of 1.5 mm for irrigation and aspiration. The view angle of the endoscope was 30°. The outer diameter of the working cannula was 7.5 mm.

#### Anesthesia and position

**Figure 1:**
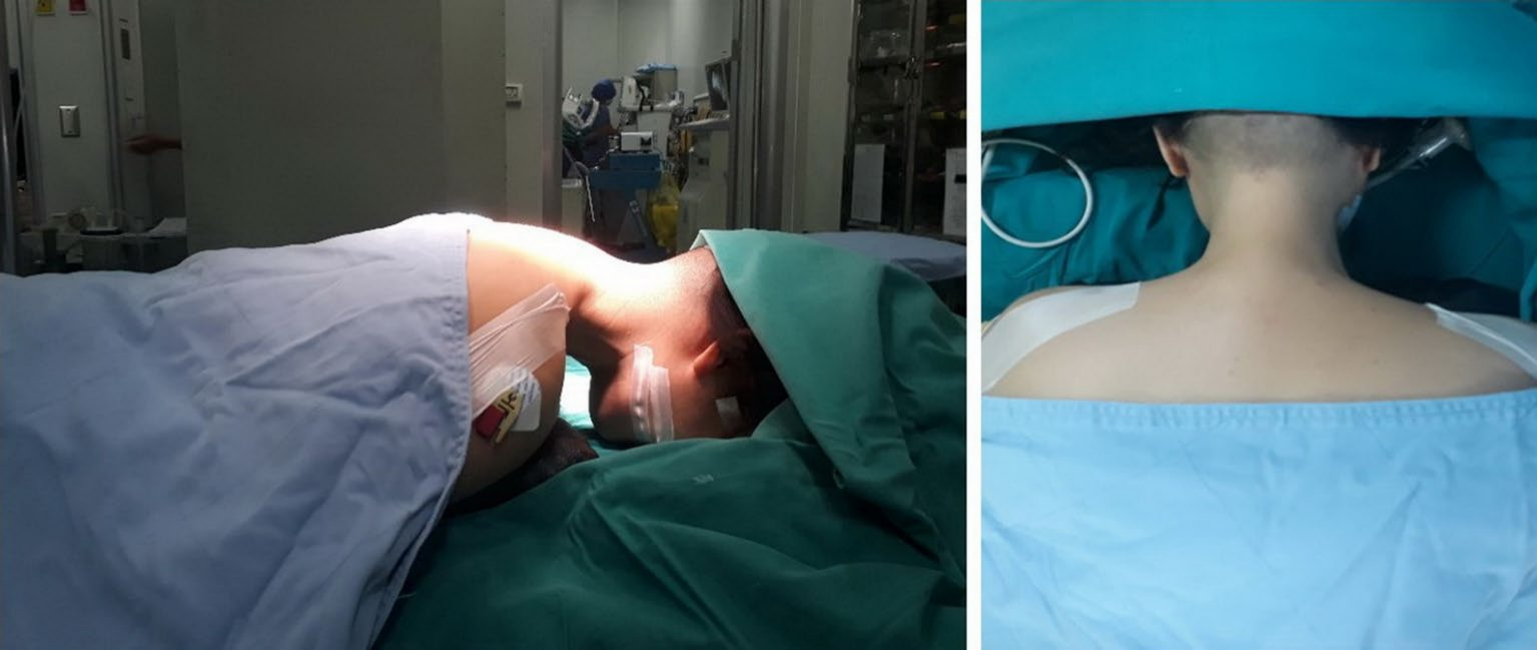
The position of a patient during surgery.

A general anesthetic was applied. The patient was positioned on the abdomen with a slightly raised head end of the operating table to reduce the pressure in the epidural venous plexus (Fig. 1). A slight straightening of the cervical lordosis was achieved for better spacing. The head was placed with a face on a soft headrest and additionally fixed to it with plaster. At that, fixation of the head with a Meinfield brace is not required. Arms were located along the torso, shoulders were shifted in caudal direction through moderate tension and fixation with the patch. This position achieves improved radiological imaging of the lower cervical segments.

The surgeon and the operating nurse were on the access side. Screens of the X-ray C-arc monitor and the endoscopic facilities were placed opposite to them.

Single intravenous antibiotics were injected just before the skin incision.

#### Approach and exposure

The line of austral spurs was highlighted with a sterile marker on the patient's skin in the area of the surgery. According to the X-ray image, the level of surgical intervention was determined in the lateral projection, and perpendicular to the median line was made. The access point was perpendicularly shifted by 1.5–2 cm from the line of pinnacles towards the pathology.

**Figure 2:**
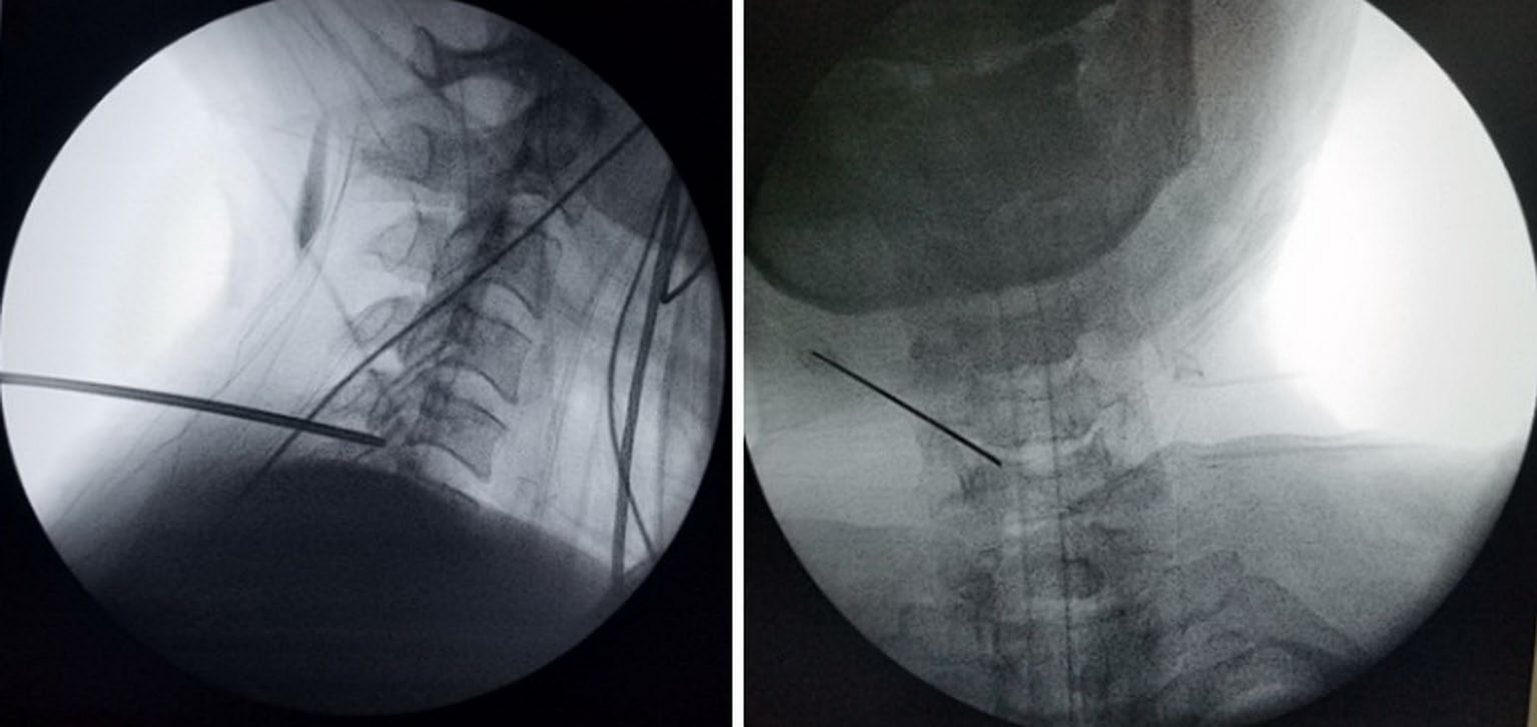
Under fluoroscopic control, the guide wires are inserted through the posterior cervical musculature with the tip directed to the operative disc space.

Under the control of fluoroscopy, a puncture needle 18G in diameter was introduced through the access point (Fig. 2), the distal end of which was placed on the lower edge of the arc of the overlying vertebrae in the area of its transition to the lower joint spine in the “V-point”^28–30^. The stylet was removed from the needle and a string conductor was inserted. The needle was taken out.

**Figure 3:**
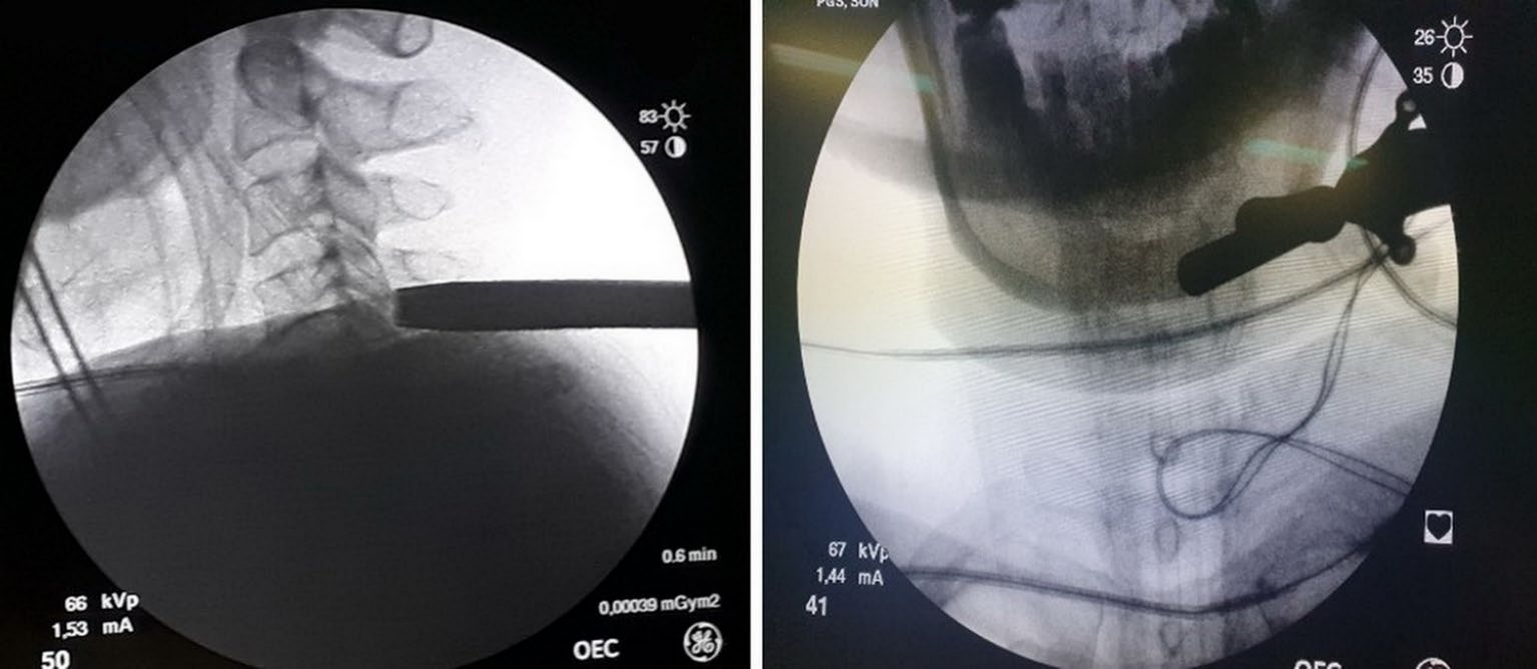
Working channel Insertion. On a string conductor, tubular reamers of increasing diameter were introduced, on which a working cannula with a beveled cut with an external diameter of 7.5 mm was installed.

The skin incision was performed with a length of 8 mm. On a string conductor, tubular reamers of increasing diameter were introduced, on which a working cannula with a beveled cut with an external diameter of 7.5 mm was installed (Fig. 3). The endoscope was injected into the cannula.

Hemostasis during the operation was achieved by regulation of pressure (about 150 cm H2O) of 0.9% sodium chloride solution supplied to the operating cavity through a special endoscope channel.

#### Laminotomy and facetectomy

**Figure 4:**
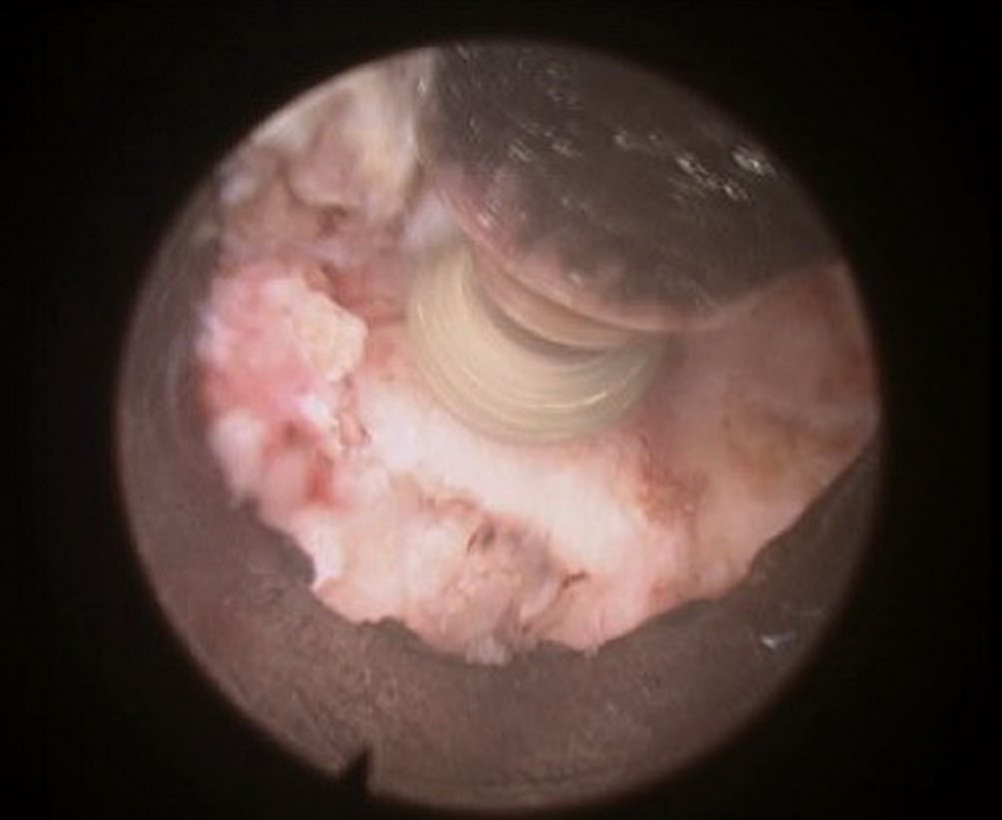
Bone Resection. Laminotomy and facetectomy are performed with 3.0 mm diamond boron, from “V-point” to the periphery.

Laminotomy and facetectomy were performed with a 3.0 mm diamond boron, from the “V-point” to the periphery (Fig. 4). The “V-point” is the main video endoscopic reference point and the center of bone resection. The facet joint is cut by no more than 50% until the lateral edge of the yellow ligament is visible. Removal of more than 50% of the facet neck joint may lead to a greater risk of further instability^31^.

Bone resection is performed with preserved integrity of the yellow ligament, which is a measure of preventing damage to the dura mater and neural structures. The formed bone window has a rounded shape with a diameter of 4–8 mm.

**Figure 5:**
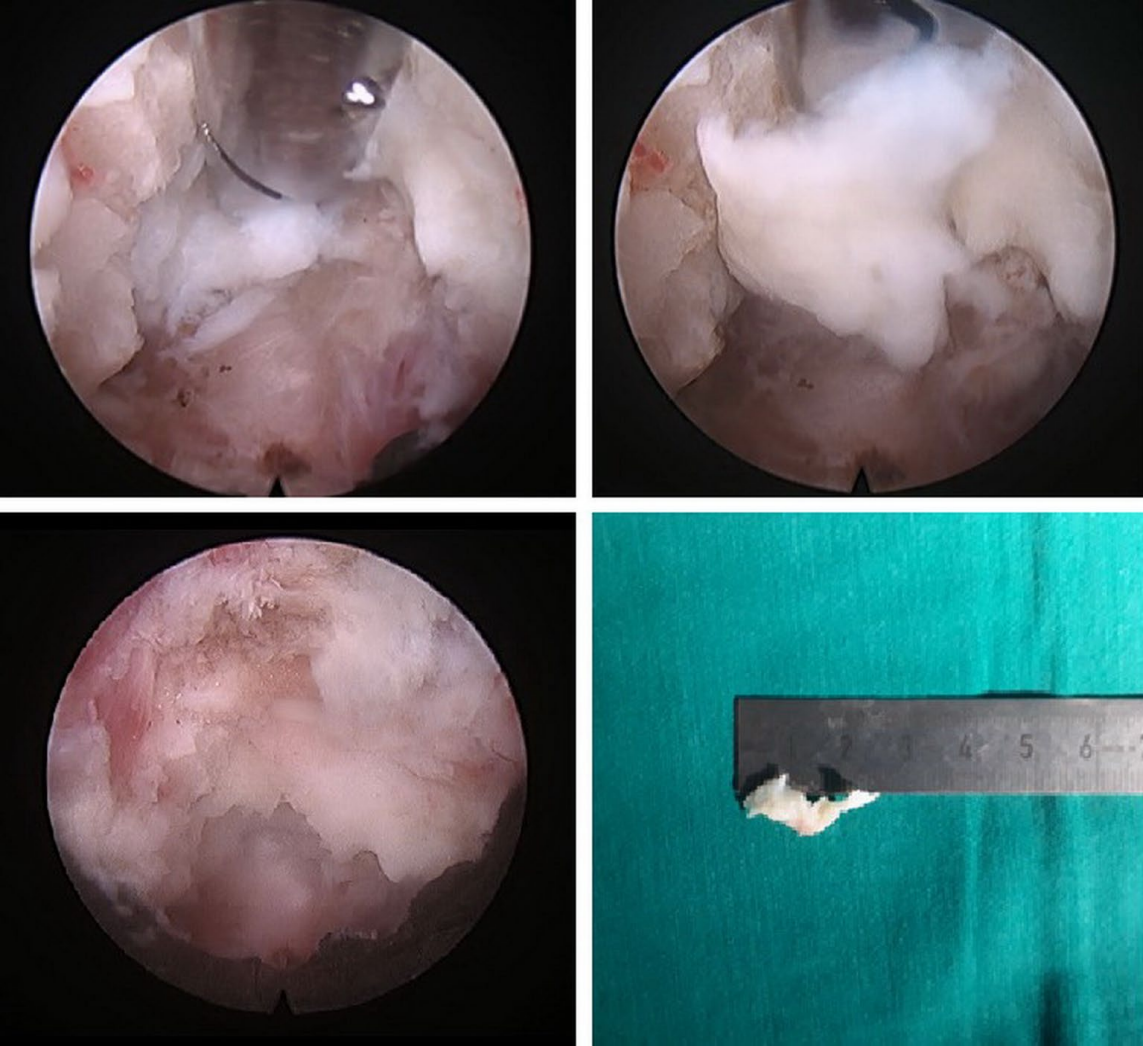
Preparation and removal of the herniated material.

**Figure 6:**
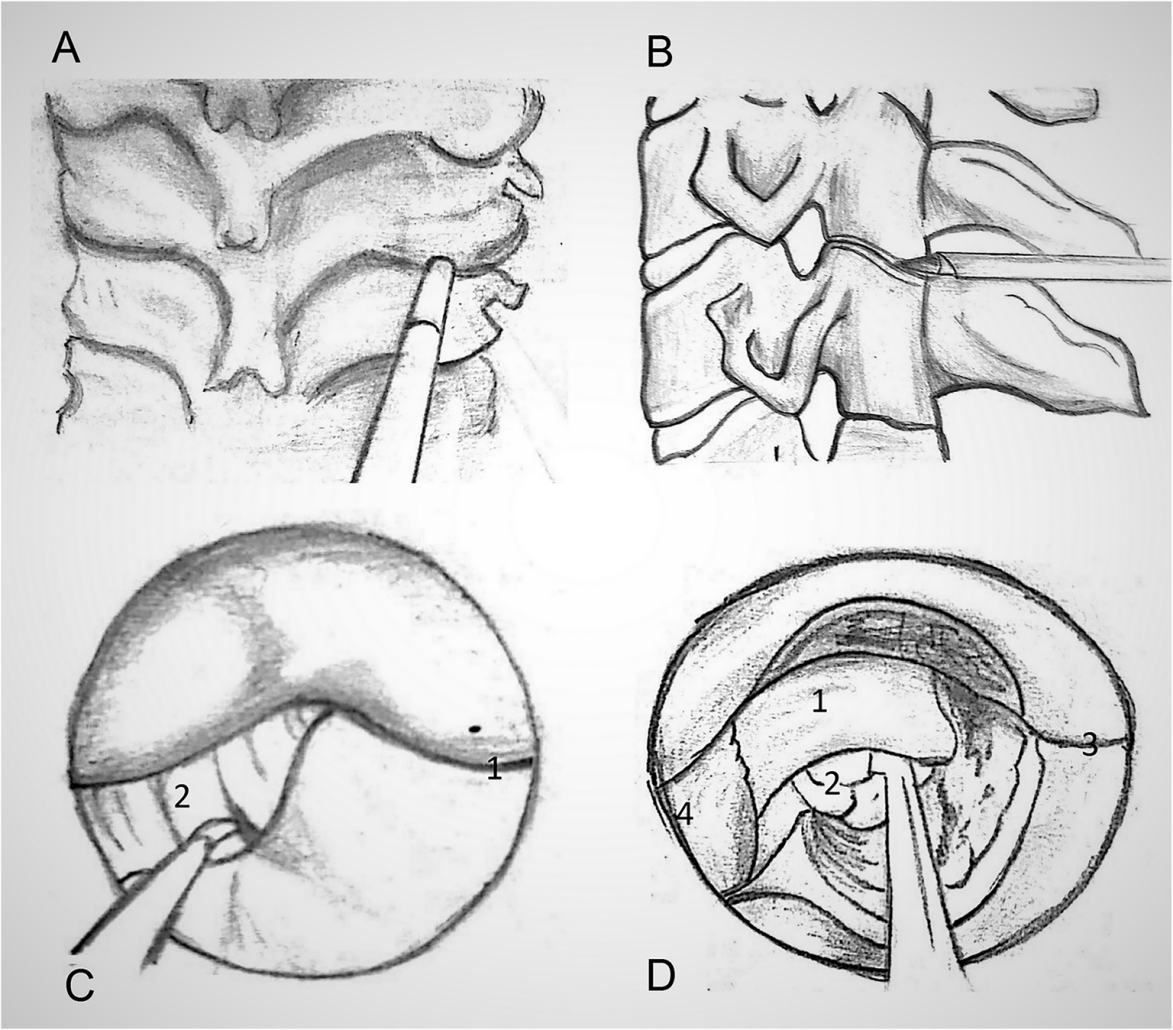
Surgical diagrams for Endoscopic Posterior Cervical Foraminotomy and Discectomy techniques: A and B localize of working cannula. C The facet joint is identified by removing the overlying soft tissue: (1) Facet joint (2) yellow ligament. D Final operative view: (1) nerve root (2) disc herniation (3) Facet joint (4) yellow ligament.

The yellow ligament was removed by Kerrison cutters, identifying the lateral edge of the dural sac and spine, located more cranial in the area of the formed bone window. The epidural plexus veins were coagulated with a bipolar radiofrequency electrode. The nerve root and intervertebral disk herniation material were mobilized with curved buttonhole and microspatula (Figs. 5 and 6).

#### Close

After removal of the hernia by cutters, the degree of decompression of the nerve roots was assessed by its transmission pulsation. The working cannula and endoscope were removed. One nodal suture is applied to the skin. No draining of the wound was required.

#### Rehabilitation

Wearing a soft cervical orthosis for seven days is recommended in the postoperative period.

### Outcome measurements

#### Visual analog scale (VAS)

The timing and degree of regression of the root pain syndrome were assessed on a VAS scale (visual and analog scale) with a comparison of preoperative and postoperative performance. The VAS is a continuous scale in the form of a horizontal or vertical line 10 cm (100 mm) long with two extreme points on it: "no pain" and "the worst pain imaginable. The patient was suggested to draw a line perpendicular across the visual analog scale to the point corresponding to the intensity of his pain. A ruler was used to measure the distance (mm) from "no pain" to "the worst pain imaginable", getting a score of 0 to 100. According to the distribution of scores, the following classification was applied: no pain (0–4 mm), mild pain (5–44 mm), moderate pain (45–74 mm), severe pain (75–100 mm). Neck pain (VAS-Neck) and arm pain (VAS-Arm) was evaluated separately.

#### Odom criteria

Evaluation of the operation outcome was carried out according to Odom criteria, representing a widely used 4-point scale for assessing clinical outcome after cervical spine surgery: excellent—no complaints referable to cervical disc disease and no functional impairment; good—intermittent discomfort without significant functional impairment; fair—subjective improvement but significant functional limitations; poor—no improvement or worsened condition. The scale was valided by a recent study.

#### Neck Disability Index (NDI)

Social adaptation of patients after surgery was assessed using the NDI (Neck Disability Index). The questionnaire consisted of 10 questions that allowed assessing the degree of social adaptation and describing the possibility of performing usual activities (work, sleep, rest, personal hygiene, lifting objects, reading, driving, headaches, concentration and pain intensity). Each question suggested 6 possible answers, and patients were asked to choose the answer that best represents their current condition. The assessment was scored on a Likert scale from 0 to 5 (0 = no functional limitation, 5 = maximum limitation). The total score ranged from 0 (no disability) to 50 (total disability). According to the distribution of scores, the following classification was applied: 0–4points (0–8%)—no disability, 5–14points (10–28%—mild disability, 15–24points (30–48%)—moderate disability, 25–34points (50–64%)—severe disability, 35–50 points (70–100%)—complete disability.

Patients were interviewed preoperatively and on days 7, 14, 21, and 28 postoperatively.

### Statistical analysis

Continuous variables are presented as means ± standard deviation. For the VAS-Neck and VAS-Arm scales, the MCID (minimum clinically important differences) was calculated using the anchor method. The MCID for VAS-Neck was 28 mm (95% confidence interval [CI] = − 32.3 to − 24.7), for VAS-Arm 26 mm (95% confidence interval [CI] = − 30.5 to − 21.6). Preoperative and postoperative pain intensity scores according to VAS-Neck and VAS-Arm scales were compared on a boxplot graph.

Statistical analysis was performed by used the program Excel for Windows.

### Ethics declarations

This study was approved by the Ethics Committee of Viet Duc University Hospital and conformed to the Helsinki Declaration.

### Consent to participate/consent to publish

All patients signed informed consent forms.

## Results

### Preoperative findings

At clinical examination and based on patients' complaints, the prevalence of brachialgia symptoms is noted, often arising after physical activity or sharp head movement. Pain had a pronounced radicular nature, was spread respectively to the dermatomes, and usually was dependent on the position of the head and extremities. Pains in the neck area, shots to the scapula, shoulder, collarbone, the spread of pain on the ‘helmet’ type or the homolateral half of the head similar to hemicrania, and dizziness were reported as well. In some cases, hypesthesia with isolated reduction of tendon reflexes from adjacent muscles was observed. Less often, the reduction of power in the proximal parts of the upper limbs was observed, being, however, unilateral and inconstant.

According to the preoperative imaging diagnostic data, the operated spinal level and the side of the pathology were established (Table 1). In most patients, the compression of the nerve roots is set at C5/C6 (65%). Multiple levers were found in one patient. The distribution of the pathology on the right and left side of the vertebrae is equal.

**Table 1:** Preoperative diagnostic imaging features (Operated spinal level and side of the pathology).

	Frequency (n)			
	C4/C5	C5/C6	C6/C7	Multiple lever
Left	0	6	4	0
Right	1	7	1	1
Total	1	13	5	1

Pain intensity values according to the VAS-neck before surgery in 15 (75%) patients were high (81.5 ± 2.1 mm), another 5 (25%) patients reported pain of moderate intensity (65.2 ± 4.2 mm). The mean pain intensity in neck of all 20 patients was 77.4 ± 7.7 mm.

According to the VAS-arm before surgery, 60% of patients reported high-intensity pain (79.2 ± 2.1 mm); another 40% of patients had pain of moderate intensity (64.5 ± 3.6 mm). The mean pain intensity in arms of all 20 patients was 73.3 ± 7.9 mm.

Three patients had nerve root palsy.

Based on preoperative NDI scores, 11 (55%) patients had complete disability (40.1 ± 2.5 points) and the remaining 9 (45%) patients had severe disability (30.2 ± 2.5 points). The average NDI score for the entire sample was 35.7 ± 5.6.

### Intraoperative indicators and postoperative outcome

The average duration of the operation was 92 ± 7.6 min. A blood loss was reported at the minimum level. Intraoperative complications, such as postoperative hematoma or infection, were not observed. None of the patients required repeated surgery in the early postoperative period.

Immediately after the operation, all patients noted regression of pain syndrome. But despite a decrease in pain intensity, 3 patients with nerve root palsy still reported high pain intensity according to VAS-neck and VAS-arm scales (76.7 ± 1.2 mm and 76.3 ± 0.6 mm, respectively). According to VAS-neck scale, pain intensity decreased to moderate (53.4 ± 66 mm) in 9 (45%) patients and mild (38.0 ± 3.3 mm) in 8 (40%) patients. The mean pain intensity score for all patients was 50.8 ± 14.2 mm. According to VAS-arm scale, 5 (25%) patients had moderate pain intensity (58.8 ± 5.3 mm) and 12 (60%) had mild pain intensity (36.4 ± 2.8 mm). The mean pain intensity index for all patients was 47.8 ± 15.8 mm. MCID was achieved in 12 (60%) patients based on VAS-neck scale (28 mm reduction in pain intensity) and also in 12 (60%) patients based on VAS-arm (26 mm reduction in pain intensity).

**Figure7:**
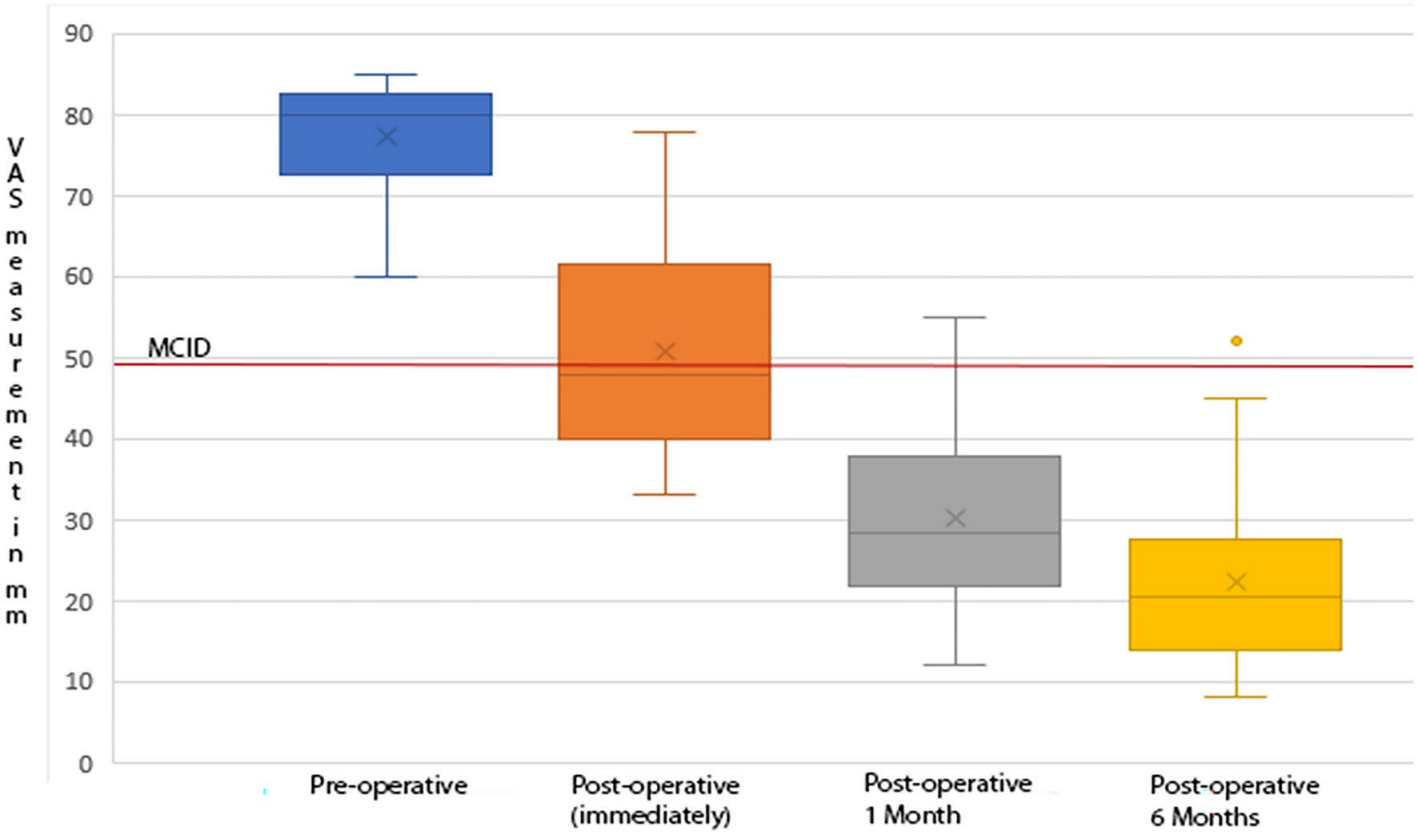
Neck pain intensity on visual analogue scale (VAS- Neck) before and after surgery. Minimal clinically important differences (MCID) = 28 mm.

**Figure 8:**
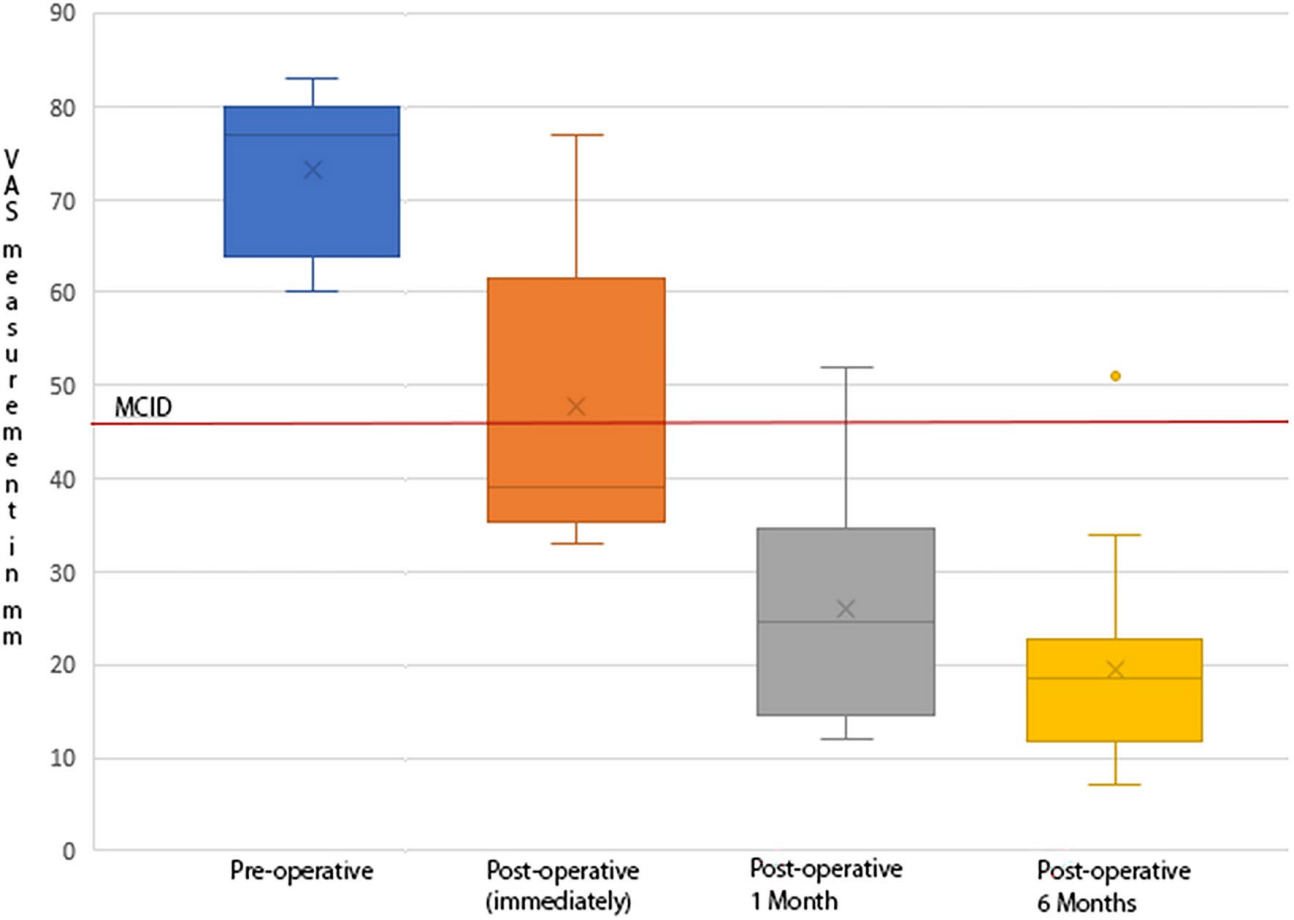
Arm pain intensity on visual analogue scale (VAS- Arm) before and after surgery. Minimal clinically important differences (MCID) = 26 mm.

When assessing the intensity of pain according to the VAS one month and six months after the operation, the tendensy to pain syndrome regression persisted. Thus, 17 (85%) of patients on VAS-neck in a month after the operation reported pain sensations of low-intensity or no pain at all (26.8 ± 8.5 mm). Another three patients (15%) had moderate intensity pain sensations (49.7 ± 4.6 mm). According to VAS-arm scale, 18 (90%) patients had pain sensations of low-intensity or no pain at all (23.4 ± 9.1 mm), while the other 2 patients (10%) suffered from moderate pain (49.5 ± 3.5 mm). The mean VAS-neck and VAS-arm pain intensity scores were 30.2 ± 11.6 and 26 ± 11.8 mm, respectively. After 6 months, pain intensity scores according to both scales demonstrated a downward trend of 22.4 ± 11.7 and 19.5 ± 10.5 mm, respectively. Only 2 patients on the VAS-neck scale and 1 patient on the VAS-arm scale retained moderate pain intensity. MCID was not achieved in 1 patient on both scales. The nerve root paralysis remained unchanged to him. The dynamics of pain intensity on VAS-neck before and after surgery is illustrated in Figs. 7 and 8.

**Figure 9:**
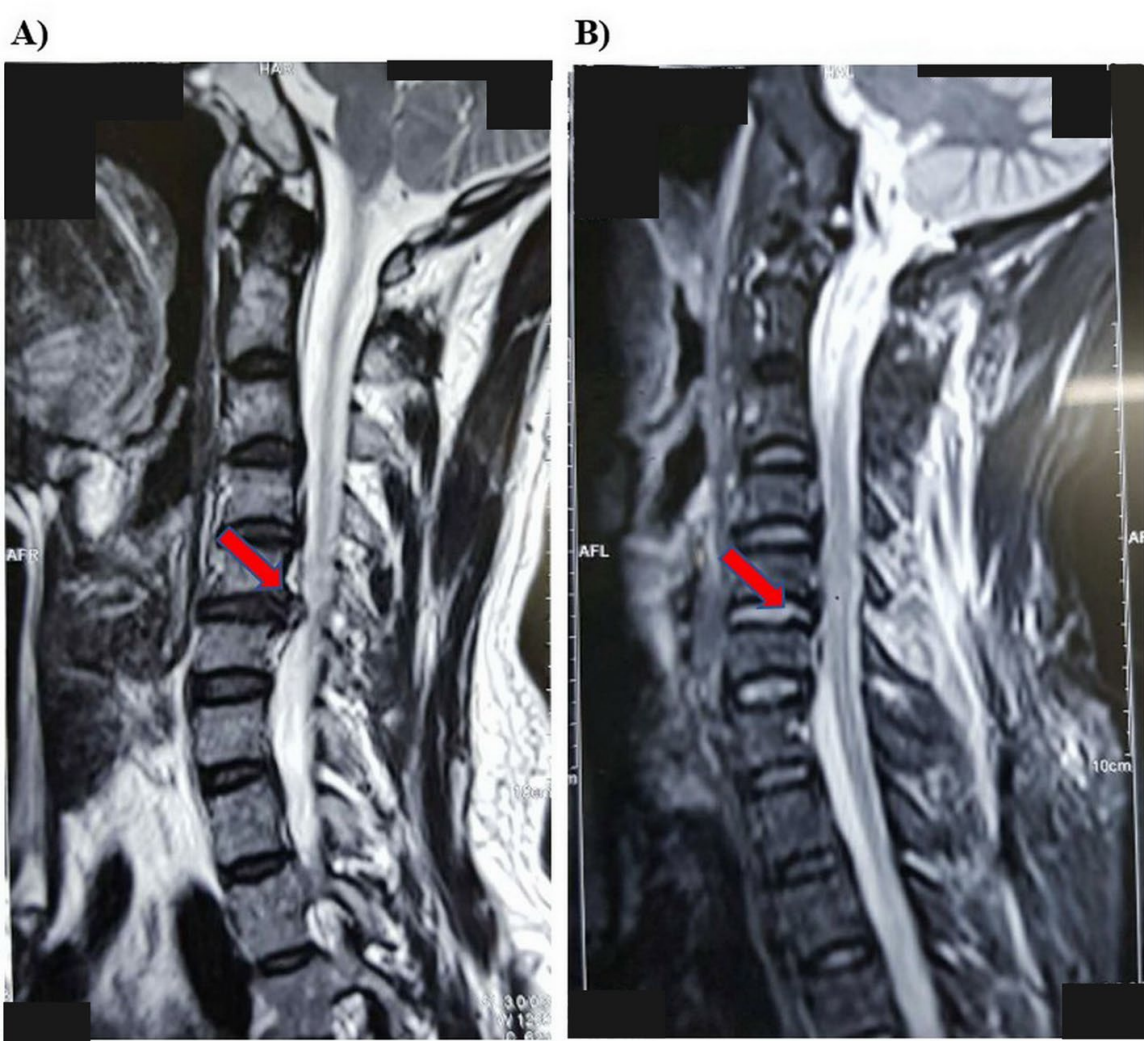
Herniation of C5-6 right. Before (**A**) and after (**B**) operation (male, 32 years old).

According to the postoperative success rate based on Odom criteria (Table 2), an excellent outcome, i.e., the disappearance of all preoperative symptoms and full clinical healing, was reported in 13 (65%) patients. In 5 patients (25%), some preoperative symptoms remained at the minimum level, which does not affect physical activity (good outcome). In 2 patients (10%), there was a reduction of preoperative symptoms with a significant limitation of physical activity (satisfactory outcome). No unsatisfactory outcomes have been noted (Fig. 9).

**Table 2:** Evaluation of surgical outcome according to Odom criteria.

The Odom criteria	Frequency (n)	Percentage (%)
Excellent	13	65.00
Good	5	25.00
Fair	2	10.00
Poor	0	0

On the NDI scale, 16 (80%) patients had only mild signs of disability or no signs at all one week after surgery. In another 3 (15%) patients, the level of mild disability was reached after 3 weeks. At the fourth week after surgery, 19 (95%) patients reported no signs of disability (Table 3). Only one patient suffered from severe disability during this period due to nerve root paralysis and persistent pain symptoms. The recovery period for this patient was up to two months.

**Table 3:** Distribution of patients according to NDI scores before and after surgery.

Classification	Pre-perative	Post-operative			
		1 week	2 week	3 week	4 week
0–4 points (no disability)	0 (0%)	6 (30%)	11 (55%)	16 (80%)	19 (95%)
5–14 points (mild disability)	0 (0%)	10 (50%)	5 (25%)	3 (15)	0 (0%)
15–24 points (moderate disability)	0 (0%)	3 (15%)	3 (15%)	0 (0%)	0 (0%)
25–34 points (severe disability)	9 (45%)	1 (5%)	1 (5%)	1 (5%)	1 (5%)
35–50 points (complete disability)	11 (55%)	0 (0%)	0 (0%)	0 (0%)	0 (0%)

## Discussion

The presented experience of fully endoscopic PCFD showed very successful results with significant reduction of preoperative symptoms, no complications, minimal blood loss, and short recovery time. These results are consistent with the experience of other authors who have applied a similar method.

In 2008, Ruetten et al.^21^ published the results of a prospective randomized comparative study of the clinical effectiveness of percutaneous video endoscopic PCFD and standard ACDF. Having analyzed the results, the authors concluded that percutaneous video endoscopic PCFD is a good alternative to standard methods of cervical discectomy in lateral soft herniated intervertebral discs with the mono-radicular syndrome. With equal clinical effectiveness, the method has advantages common to mini-invasive procedures.

Recently, Wan et al.^32^ reported on the experience of performing full-endoscopic PCFD under local anesthesia. A total of 25 patients were operated on. Preoperative symptoms were reported to disappear in 24 patients, while no serious complications were observed in any of the patients.

A study by Shohin et al.^33^ examined the recovery of motor strength after full-endoscopic PCFD and reported an improvement in preoperative motor weakness in 95% of patients and a full recovery of motor function in 86% of patients one year after surgery. Excellent and good outcome of minimally invasive percutaneous cervical discectomy surgeries in 82% of the cases was also reported in the literature^6^.

Ohmori et al.^34^ compared the results of full-endoscopic PCFD application for surgical treatment of lateral hernia of the intervertebral disc in the cervical spine and spondylotic foraminal stenosis and established their equivalence.

The results of other studies show that the method of full-endoscopic cervical foraminotomy and discectomy provides comparable results, similar to the frequency of complications and repeated operations, but with less blood loss, less intensity of pain, faster recovery, and shorter hospital stay in comparison with the method of open cervical foraminotomy and discectomy^17,18^. It also reduces pain during postoperative recovery, the probability of potential wound complications, and the risk of postoperative kyphosis caused by the removal of posterior cervical muscles.

The choice of surgical approaches, including full-endoscopic, in the treatment of compression syndromes represented by neurological disorders, is associated with some controversial issues^6,35^. The main question is in the selection of anterior and posterior decompression options.

Yang et al.^6^ compared the results of full-endoscopic ACDF and PCFD and reported no significant differences in clinical results between the two approaches. At the same time, the authors noted that full-endoscopic PCFD may be more preferable to ACDF given the volume of disk removal, duration of hospital stay, and postoperative radiological changes of the cervical spine in dynamics. In a study by Kim and co-authors^28^, full-endoscopic PCFD was proven to have no effect on the sagittal balance of the cervical spine.

Both methods allow realizing the set task in full enough. The choice of the decompression direction is determined by the localization of the compression factor, which has been proved in single-level interventions. Modern neuroimaging methods enable determining the nature of the compression factor: ‘soft’ (herniation of the intervertebral disk) or ‘hard’ (osteophite or bone spike), which is decisive in the choice of surgical access. In the case of a ‘soft’ lateral or intraforaminal disk herniation and foraminal stenosis and lateral recess stenosis, full-endoscopic PCFD is evident, while osteophyte in this area can be more radically eliminated by a ventral approach^19^.

The findings of this research are well consistent with the above-described concepts and complement the already known favorable studies of endoscopic approaches.

However, the need for a prospective large-scale examination by comparing full-endoscopic techniques with traditional methods is indisputable. Such a study will help to better define the role of endoscopic approaches compared to conventional cervical accesses in the treatment of cervical radiculopathy.

## Conclusions

The results obtained confirmed the safety and efficiency of full-endoscopic PCFD at soft lateral or intraforaminal disk herniation, as well as foraminal stenosis and lateral recess stenosis. With correctly chosen evidence for surgery, the clinical results of this type of operational intervention correspond to the previously described open and minimally invasive methods of treatment. At the same time, the described surgical technique provides much less tissue traumatization while accessing the operational area. Thus, the time a patient spends in the hospital reduces, and his or her ability to work restores quite fast as well. Also, a decrease in cases of postoperative local pain or muscle spasm and the need for pain meds in the postoperative period are reported. Full-endoscopic PCFD is a worthy alternative to open surgery methods and fits into the general concept of modern spinal surgery development. The main disadvantages are the complexity of training in endoscopic methods for surgeons accustomed to traditional open access, the limited direct field of vision, and a narrow working channel. The prospects for the treatment of discogenic compression syndromes at the cervical level are undeniable. However, due to the high-risk profile of the cervical spine, caution is required in such approaches.

## Data Availability

Data will be available on request.

## References

[1] Gushcha AO, Arestov SO, Vershinin AV (2016). Early experience of using the new technique of portal endoscopic discectomy for intervertebral disk herniations of the cervical spine. Voprosy Neirokhirurgii im. N.N. Burdenko.

[2] Woods BI, Hilibrand AS (2015). Cervical radiculopathy: Epidemiology, etiology, diagnosis, and treatment. J. Spinal. Disord. Tech..

[3] Lawrence JC (1969). Disc degeneration: Its frequency and relationship to symptoms. Ann. Rheum. Dis..

[4] Rhee JM, Yoon T, Riew KD (2007). Cervical radiculopathy. J. Am. Acad. Orthop. Surg..

[5] Fehlings MG, Gray RJ (2009). Posterior cervical foraminotomy for the treatment of cervical radiculopathy. J. Neurosurg. Spine.

[6] Yang JS (2014). Anterior or posterior approach of full-endoscopic cervical discectomy for cervical intervertebral disc herniation? A comparative cohort study. Spine.

[7] Clarke MJ, Ecker RD, Krauss WE, McClelland RL, Dekutoski MB (2007). Same-segment and adjacent-segment disease following posterior cervical foraminotomy. J. Neurosurg. Spine.

[8] Liu WJ, Hu L, Chou PH, Wang JW, Kan WS (2016). Comparison of anterior cervical discectomy and fusion versus posterior cervical foraminotomy in the treatment of cervical radiculopathy: A systematic review. Orthop. Surg..

[9] Witiw CD, Smieliauskas F, O'Toole JE, Fehlings MG, Fessler RG (2019). Comparison of anterior cervical discectomy and fusion to posterior cervical foraminotomy for cervical radiculopathy: Utilization, costs, and adverse events 2003 to 2014. Neurosurgery.

[10] Roh SW, Kim DH, Cardoso AC, Fessler RG (2000). Endoscopic foraminotomy using MED system in cadaveric specimens. Spine.

[11] Burke TG, Caputy A (2000). Microendoscopic posterior cervical foraminotomy: A cadaveric model and clinical application for cervical radiculopathy. J. Neurosurg..

[12] Winder MJ, Thomas KC (2011). Minimally invasive versus open approach for cervical laminoforaminotomy. Can. J. Neurol. Sci..

[13] Ross DA, Bridges KJ (2017). Technique of minimally invasive cervical foraminotomy. Oper Neurosurg..

[14] Ruetten S, Komp M, Godolias G (2006). A new full-endoscopic technique for the interlaminar operation of lumbar disc herniations using 6-mm endoscopes: Prospective 2-year results of 331 patients. Minim. Invasive. Neurosurg..

[15] Lawton CD (2014). Clinical outcomes of microendoscopic foraminotomy and decompression in the cervical spine. World Neurosurg..

[16] Ruetten S, Komp M, Merk H, Godolias G (2007). Use of newly developed instruments and endoscopes: Full-endoscopic resection of lumbar disc herniations via the interlaminar and lateral transforaminal approach. J. Neurosurg. Spine.

[17] Zhang C, Wu J, Zheng W, Li C, Zhou Y (2020). Posterior endoscopic cervical decompression: Review and technical note. Neurospine.

[18] Achmad H, Sarina BD, Ramadhany YF, Kirichenko EV, Markov A (2020). The impact of using antibiotic drugs in pediatric dentistry. Int. J. Pharm. Res..

[19] Arestov SO, Vershinin AV, Gushcha AO (2014). Comparison of the effectiveness and capabilities of endoscopic and microsurgical methods for removing herniated intervertebral discs of the lumbosacral spine. Neurosurg. Issues.

[20] Ruetten S, Komp M, Merk H, Godolias G (2007). A new full-endoscopic technique for cervical posterior foraminotomy in the treatment of lateral disc herniations using 6.9-mm endoscopes: Prospective 2-year results of 87 patients. Minim. Invasive Neurosurg..

[21] Ruetten S, Komp M, Merk H, Godolias G (2008). Full-endoscopic cervical posterior foraminotomy for the operation of lateral disc herniations using 5.9-mm endoscopes: A prospective, randomized, controlled study. Spine.

[22] Kim CH, Chung CK, Kim HJ, Jahng TA, Kim DG (2009). Early outcome of posterior cervical endoscopic discectomy: An alternative treatment choice for physically/socially active patients. J. Korean Med. Sci..

[23] Kim CH (2015). Minimally invasive cervical foraminotomy and diskectomy for laterally located soft disk herniation. Eur. Spine J..

[24] Kravtsov MN, Lyulin SV, Kuznetsov MV, Gaidar BV, Svistov DV (2018). Percutaneous video endoscopic posterior cervical foraminotomy and discectomy for lateral herniated intervertebral discs (literature review and results own research). Genius Orthop..

[25] Gladilina IP, Yumashev AV, Avdeeva TI, Fatkullina AA, Gafiyatullina EA (2018). Psychological and pedagogical aspects of increasing the educational process efficiency in a university for specialists in the field of physical education and sport. Espacios.

[26] Ye ZY (2017). Clinical observation of posterior percutaneous full-endoscopic cervical foraminotomy as a treatment for osseous foraminal stenosis. World Neurosurg..

[27] Dion S, Stupar M, Côté P, Grenier JM, Taylor JA (2018). Criteria to screen for traumatic cervical spine instability: A consensus of chiropractic radiologists. J. Manip. Physiol. Ther..

[28] Kim CH, Shin KH, Chung CK, Park SB, Kim JH (2015). Changes in cervical sagittal alignment after single-level posterior percutaneous endoscopic cervical discectomy. Global Spine J..

[29] Moses MJ (2019). Comparison of patient reported outcome measurement information system with neck disability index and visual analog scale in patients with neck pain. Spine.

[30] Broekema AEH (2019). The Odom criteria: Validated at last: A clinimetric evaluation in cervical spine surgery. J. Bone Joint Surg. Am..

[31] Uthaikhup S, Paungmali A, Pirunsan U (2011). Validation of Thai versions of the Neck Disability Index and Neck Pain and Disability Scale in patients with neck pain. Spine.

[32] Wan Q (2018). Posterior percutaneous full-endoscopic cervical discectomy under local anesthesia for cervical radiculopathy due to soft-disc herniation: A preliminary clinical study. J. Neurosurg. Spine..

[33] Shohin IE, Bagaeva NS, Malashenko EA, Kuzina VN (2020). Method of estimating the equivalence of dissolution profiles: A modern view. Drug Dev. Regist..

[34] Ohmori K, Ono K, Hori T (2017). Outcomes of full-endoscopic posterior cervical foraminotomy for cervical radiculopathy caused by bony stenosis of the intervertebral foramen. Mini-invasive Surg..

[35] Li XC, Zhong CF, Deng GB, Liang RW, Huang CM (2016). Full-endoscopic procedures versus traditional discectomy surgery for discectomy: A systematic review and meta-analysis of current global clinical trials. Pain Physician.

